# Presoaking with Sodium Selenite Promotes Accumulation of Polyphenols and GABA in Foxtail Millet Sprouts Under NaCl Stress

**DOI:** 10.3390/foods15101778

**Published:** 2026-05-18

**Authors:** Huiying Fu, Shuaiduo Sun, Yaoxi Liu, Guowei Man, Junjie Hao, Jinle Xiang

**Affiliations:** 1 Faculty of Food & Bioengineering, Henan University of Science & Technology, Luoyang 471023, China; 15936082124@163.com (H.F.); 15037912413@163.com (S.S.); peach90@yeah.net (G.M.); 2 Department of Tourism and Food, Henan Forestry Vocational College, Luoyang 471000, China; hnlxlyx@126.com; 3 Institute of Plant Protection, Henan Academy of Agricultural Sciences, Zhengzhou 450002, China; haojjds@163.com; 4 Henan Key Laboratory of Agricultural Product Processing Technology, Henan University of Science & Technology, Luoyang 471023, China

**Keywords:** foxtail millet sprouts, selenium enrichment, salt stress, polyphenols, GABA, response surface methodology

## Abstract

The effects of presoaking with sodium selenite (Na_2_SeO_3_) solution on the polyphenols and *γ*-aminobutyric acid (GABA) in foxtail millets during germination under NaCl stress condition were investigated, and the key processing parameters, including Na_2_SeO_3_ concentration, presoaking time, presoaking temperature and NaCl concentration, were optimized via response surface methodology (RSM) based on total phenolic content (TPC) and GABA content of foxtail millet sprouts. The inhibition of sprout growth caused by salt stress was alleviated by presoaking with Na_2_SeO_3_, which did not alter phenolic compositions, resulting in a significant increase in the levels of both phenolics and GABA. The optimal germination parameters were 42 mg/L Na_2_SeO_3_, 9.8 h soaking time, 30 °C soaking temperature, and 110 mmol/L NaCl. Under these conditions, the measured TPC and GABA content were 837.22 mg FAE/100 g and 281.68 mg/kg, respectively, which closely approximated the predicted values. Correspondingly, the main free phenolic compounds 3-*p*-coumaroylquinic acid and *N*-*p*-coumaroylserotonin increased by 2.94 and 3.34 times, respectively, and the predominant bound phenolic compounds *trans*-ferulic acid and *trans*-*p*-coumaric acid increased by 2.28 and 6.39 times, respectively. Meanwhile, the total and organic selenium contents of the sprouts reached 14.74 and 12.02 mg/kg dry weight, respectively. This study provides a practical technology for preparing selenium-enriched foxtail millet sprouts with enhanced phenolic compounds and GABA, which can serve as a novel functional food resource.

## 1. Introduction

As one of the earliest domesticated cereals in human history, foxtail millet (*Setaria italica* (L.) P. Beauv) is valued for its nutritional and health-promoting properties, which stem from its rich array of bioactive constituents [1]. Polyphenols are one of the main bioactive components in foxtail millet and are widely recognized for their various health-promoting properties, including antioxidant, anti-inflammatory, cardioprotective, neuroprotective, antidiabetic, anti-cancer, and anti-aging effects [2,3,4]. GABA is another important secondary metabolite with multiple health benefits. GABA plays a crucial role in signal transmission, regulation of neuronal development, and the improvement of sleep and mood as the principal inhibitory neurotransmitter in the human nervous system [5]. GABA also exhibits other important health benefits, including antihypertensive, antidiabetic, anti-inflammatory, and antidepressant properties [6]. Germination has been proven to be an effective means to improve cereal quality by enhancing the levels of polyphenols and GABA, while reducing antinutritional factors, for example, phytic acid in foxtail millet [7]. Foxtail millet sprouts are expected to be explored as a valuable food material with both health-promoting and nutritional value.

The functional food value of foxtail millet sprouts can be improved by optimizing germination conditions to increase their bioactive compound content. Some abiotic stresses, such as salinity, low temperature, and drought, have been demonstrated to significantly enhance secondary metabolite biosynthesis during seed germination [8]. In particular, salt stress has been proven to enhance the activity of related enzymes in germinated hulless barley seeds, thereby promoting the accumulation of phenolic compounds [9]. Although foxtail millet generally exhibits salt tolerance, it remains highly sensitive to salt stress during the germination stage [10]. Excessive salinity results in a decrease in relative water content and lipid peroxidation, thereby inhibiting the growth of foxtail millet sprouts [11].

Our previous work has revealed that low concentrations of Na_2_SeO_3_ presoaking treatment significantly boosted the germination rate, GABA and phenolic compound synthesis, and antioxidant capacity of foxtail millet sprouts [12]. Selenium (Se) has been proven to enhance the activities of various antioxidant enzymes, thereby mitigating the adverse effects of abiotic stress in plants [13]. Se application has been reported to alleviate salt stress by breaking seed dormancy and enhancing vigor, which in turn promotes germination and seedling growth [14]. Se could be effectively enriched by seed germination, and moderate Se intake plays a key role in antioxidant defense, thyroid hormone metabolism, and immune function, whereas excessive intake may lead to selenosis and potential thyroid dysfunction [15]. Given the recognized connection between Se and plant responses to abiotic stress, it is putatively considered that the growth and secondary metabolite profiles of foxtail millet sprouts under salt stress may be modified by suitable Na_2_SeO_3_ treatment.

However, there is limited research on the impact of Na_2_SeO_3_ presoaking treatment on cereal grain biochemistry under NaCl stress. Our previous study investigated the influences of Se-enriched germination by Na_2_SeO_3_ presoaking on the growth performance and main functional components in foxtail millet sprouts [12]. In addition, it has been confirmed that some bioactive phytochemicals, such as GABA and phenolics, could be boosted by germination under salt stress [16,17]. In the present study, Na_2_SeO_3_ presoaking combined with NaCl stress germination was conducted to investigate the influence of Se-enriched treatment on sprout growth and the accumulation of polyphenols and GABA under salt stress. The primary objectives of this research were to: (1) investigate the effects of Na_2_SeO_3_ presoaking treatment on the growth and bioactive components in foxtail millet sprouts under different NaCl concentrations, and (2) optimize germination conditions using RSM to enhance phenolic and GABA yields in foxtail millet sprouts. This study established a practical germination protocol to significantly enrich both Se and bioactive compounds in foxtail millet sprouts, thereby transforming them into high-value functional food material.

## 2. Materials and Methods

### 2.1. Materials and Chemicals

The foxtail millet seeds (Jinsu NO1) were supplied by Henan Jinsu Agricultural Technology Co., Ltd. (Luoyang, China). Na_2_SeO_3_, chromatographic grade cyclohexane, and 2,3-Diaminonaphthalene (DAN) were procured from Tianjin Chemical Reagent Factory Co., Ltd. (Tianjin, China), Tianjin Yongda Chemical Reagent Co., Ltd. (Tianjin, China), and Shanghai Macklin Biochemical Technology Co., Ltd. (Shanghai, China), respectively. Chromatographic grade acetonitrile, formic acid, and methanol were products of Thermo Fisher Scientific Reagent Co., Ltd. (Waltham, MA, USA). Folin–Ciocalteu reagent was procured from Sigma-Aldrich (St. Louis, MO, USA). Chemical standards, including syringic acid, *p*-hydroxybenzaldehyde, vanillic acid, *p*-hydroxybenzoic acid, *p*-coumaric acid, and *γ*-aminobutyric acid (GABA), were supplied by Shanghai Yuanye Biotechnology Co., Ltd. (Shanghai, China). Analytical grade reagents, including sodium hydroxide (NaOH), sodium carbonate (Na_2_CO_3_), n-hexane and ethyl acetate, were acquired from Tianjin Dean Chemical Reagent Co., Ltd. (Tianjin, China), while sodium chloride (NaCl) was sourced from Enox Co., Ltd. (Jiangsu, China).

### 2.2. Germination Process of Foxtail Millet

The foxtail millet seeds were presoaked for 12 h at 25 °C in either 20 mg/L Na_2_SeO_3_ solution or pure water. Subsequently, the seeds were placed evenly in Petri dishes and incubated at 25 °C for a fixed germination time of 3 days. During the germination period, pure water was uniformly sprayed every 12 h on the first day, and an equal volume of NaCl solution at different concentrations (0, 10, 50, 100, 200 mM) was sprayed every 12 h for the following two days. Then the foxtail millet sprouts were collected, freeze-dried, and ground into powder for analysis.

### 2.3. Measurement of Sprout Length

Thirty foxtail millet sprouts were randomly selected from each germination dish, and the sprout length was measured using a ruler. The average length was calculated and expressed as mm per sprout.

### 2.4. Optimization of Germination Conditions

#### 2.4.1. Single-Factor Experiments

The impacts of six factors, including Na_2_SeO_3_ concentration (0–100 mg/L), soaking time (4–14 h), soaking temperature (20–40 °C), NaCl concentration (0–250 mM), germination time (3–7 d), and germination temperature (15–35 °C), were examined for total phenolic content (TPC) and GABA content of foxtail millet sprouts via single-factor experiments.

#### 2.4.2. Response Surface Methodology (RSM) Using Box–Behnken Design (BBD)

Based on single-factor experimental results, a four-factor, three-level Box–Behnken design (BBD) combined with response surface methodology (RSM) was employed to optimize the key variables influencing TPC and GABA levels in foxtail millet sprouts. The four factors, Na_2_SeO_3_ concentration (A), soaking time (B), soaking temperature (C), and NaCl concentration (D), were considered through the single-factor experiments. Three independent variables were assigned three levels (−1, 0, 1) as presented in Table 1. The coefficients of the quadratic polynomial model were then determined using Design Expert 13.0.0 (Stat-Ease Inc., Minneapolis, MN, USA). The GABA content and TPC were used as response values Y1 and Y2, respectively. A total of 29 experimental groups were designed, as shown in Table 2. The adequacy of the fitted model was evaluated using the coefficient of determination (R^2^), while its statistical significance was assessed using the F-test and P-test. Analysis of variance (ANOVA) was used to perform statistical analysis of the established model. Regression coefficients were obtained by fitting experimental data into the quadratic polynomial model (Equation (1)).

(1) $$\gamma =\beta_{0}+\sum_{i=1}^{k}\beta_{i}x_{i}+\sum_{i=1}^{k}\beta_{\mathrm{ii}}x_{i}^{2}+\sum_{i}^{k-1}\sum_{j}^{k}\beta_{\mathrm{ij}}x_{i}x_{j}$$

where γ denotes the response variable (GABA and TPC), x_i_ and x_j_ are the independent variables, β_0_ is the constant term, β_i_ and β_ii_ are the linear and quadratic coefficients, respectively, and β_ij_ represents the interaction coefficient.

### 2.5. Determination of Selenium (Se) Content

Total Se and inorganic Se contents were determined according to our previously reported fluorescence spectrophotometry [18]. Briefly, sample powder (0.5 g) was thermally digested in 10 mL of 9:1 (*v*/*v*) HNO_3_ and HClO_4_ mixture over 12 h, then supplemented with 5 mL of 6 M HCl. After evaporating the solution to approximately 2 mL, the DAN reagent was introduced, and then the mixture was heated for 5 min. Following extraction with cyclohexane, the fluorescence intensity of the organic phase was measured using a fluorescence spectrophotometer (Cary Eclipse; Agilent Instruments, Santa Clara, CA, USA), and the total Se content was quantified as milligrams per kilogram (mg/kg DW).

Inorganic Se in the extract was then quantified. Briefly, 0.2 g of the sample powder was accurately weighed, and 20 mL of ultrapure water was added. After 30 min of ultrasound-assisted extraction, the mixture was centrifuged at 10,000 rpm for 10 min. The supernatant obtained was designated as the inorganic Se extract. Subsequent operations were performed following the same procedure as previously described for the determination of the total Se content. The organic Se content was determined by subtracting the inorganic Se content from the total Se content.

### 2.6. Extraction and Determination of Polyphenols

The extraction of phenolic compounds followed the method described by Xiang et al. [19]. The TPC was measured following the method described by Zheng et al. [20]. Briefly, 10 μL of polyphenol extract was combined with 40 μL of Folin–Ciocalteu reagent in a 96-well microplate. Then 160 μL of Na_2_CO_3_ solution was added to each well, and the reaction mixture was incubated in darkness at room temperature for 1.5 h. Absorbance was measured at 750 nm, and the results were expressed as milligrams of ferulic acid equivalent per 100 g of dry weight (mg FAE/100g DW). The TPC of the foxtail millet sprouts was calculated by summing the free and bound phenolic content values.

### 2.7. UPLC-MS/MS Conditions and Quantification of Individual Phenolic Compounds

Separation and quantification of phenolic compounds from foxtail millet sprout extracts were achieved via UPLC-MS/MS (Waters UPLC H-Class, Waters Xevo TQ-S/micro, Waters Corporation, Milford, MA, USA). An Accucore C_18_ column (100 mm × 3 mm, Thermo Fisher Scientific, Waltham, MA, USA) was employed as the analytical column. The injection volume was 3 μL, with a column temperature of 25 °C and a flow rate of 0.4 mL/min. A binary mobile phase system consisting of (A) 0.1% formic acid in ultrapure water and (B) 0.1% formic acid in methanol was employed. The gradient elution program and mass spectrometer parameters were set according to our previously established method [21]. The external standard method was used to calculate the content of each phenolic compound. For phenolic compounds without available standards, quantification was performed using the concentration equivalent of their closest structural match. The concentrations of target compounds in foxtail millet sprouts were reported as milligrams per kilogram of dry weight (mg/kg DW).

### 2.8. Extraction and Determination of GABA

As described for the free phenolic extraction, the ground sample was defatted twice with n-hexane, then mixed with 80% methanol solution and shaken in the dark for 1 h. After centrifugation, the supernatant was collected. The extraction process was repeated once, and all supernatants were combined to obtain the GABA extract. GABA was quantified using UPLC-MS/MS, and the analysis column and UPLC/MS equipment were identical to those described above. Binary mobile phases A and B were 0.1% formic acid solution and acetonitrile, respectively. A 10 μL sample was injected, and linear gradient elution was carried out at a flow rate of 0.2 mL/min. The detailed elution gradient was set as follows: 0 min for 5% B, 3 min for 90% B, and 6–8 min for 5% B. Mass spectrometric detection was performed in positive ion (ESI^+^) mode using multiple reaction monitoring (MRM). Other parameters were set following the method previously established by Li et al. [22]. Using 104.0/87.0 (*m*/*z*) as a quantitative ion pair, the content of GABA in the millet sprouts was quantitatively analyzed by the external standard method. Results for GABA were presented as milligrams per kilogram of dry weight (mg/kg DW).

### 2.9. Statistical Analysis

All experiments were performed in triplicate, and the data were expressed as mean ± standard deviation (SD). Differences among groups were evaluated by Tukey’s multiple comparison test using SPSS 27.0 (IBM Corp., Armonk, NY, USA), with *p* < 0.05 considered statistically significant. The response surface was designed and analyzed by Design Expert 13.0 software. ANOVA, incorporating the lack-of-fit test, coefficient of determination (R^2^), and F-test (*p* < 0.05), was employed to evaluate model adequacy.

## 3. Results and Discussion

### 3.1. Effects of Na_2_SeO_3_ on the Growth of Foxtail Millet Sprouts Under NaCl Stress

**Figure 1:**
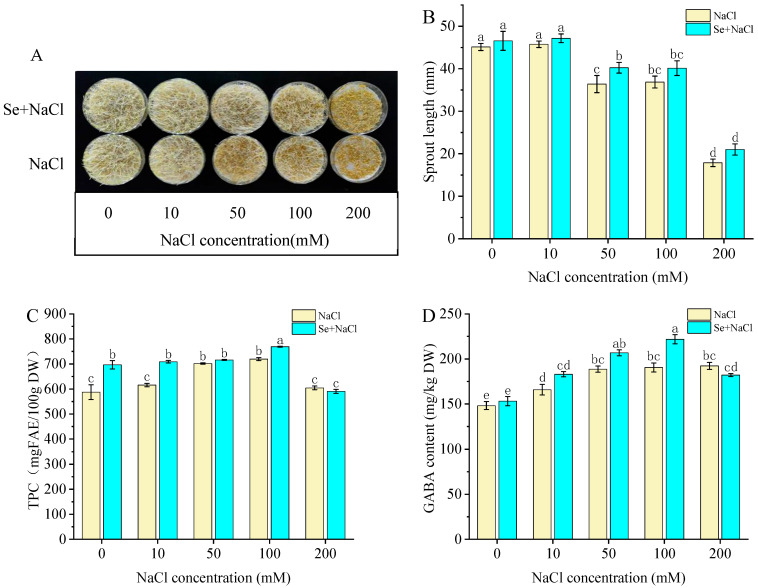
Effects of NaCl stress on the growth (**A**), sprout length (**B**), TPC (**C**), and GABA content (**D**) of foxtail millet sprouts with or without Na_2_SeO_3_ presoaking. Different lowercase letters indicate significant differences between treatment groups (*p* < 0.05). Note, NaCl: NaCl stress treatment; Se: Na_2_SeO_3_ presoaking treatment; Se + NaCl: group subjected to both NaCl stress and Na_2_SeO_3_ presoaking treatment. (**A**) Pale yellowish-white indicates well-grown sprouts, yellowed indicates ungerminated seeds or sprouts severely inhibited by NaCl stress.

Growth morphology and sprout length have been identified as sensitive indicators of plant stress levels, which can directly reflect the degree of stress [23]. The impact of different NaCl concentrations on foxtail millet sprout growth and length is depicted in Figure 1A,B, respectively. In Figure 1A, pale yellowish-white elongated sprouts and radicles represent well-grown foxtail millet sprouts, while yellowed indicates ungerminated seeds or sprouts severely inhibited by NaCl stress. The growth of foxtail millet sprouts was obviously inhibited when the NaCl concentration exceeded 50 mM, with the inhibitory effect intensifying progressively at higher concentrations. However, the stress effect exerted by NaCl on foxtail millet sprouts could be alleviated by presoaking with Na_2_SeO_3_ solution. When the NaCl concentration was 50 mM, the sprouts were 10.5% longer than the control (*p* < 0.05).

High salinity inhibits plant growth through disruption of the cellular homeostasis of water potential and ion distribution [24]. Furthermore, the delicate balance between the production and scavenging of reactive oxygen species (ROS) is disrupted under NaCl stress. ROS, including hydroxyl radicals, superoxide anions, and hydrogen peroxide, are byproducts of normal plant cellular metabolism. Excessive accumulation of ROS causes oxidative damage to cellular components and ultimately inhibits the growth of sprouts [25]. Exogenous Se was considered beneficial to the growth and development of plant seeds, because Se at low concentrations can act as an antioxidant protector for plants [14]. Se was believed to mitigate salt-induced oxidative stress damage by inhibiting the absorption of Na^+^ in turnip seeds and stimulating proline biosynthesis, thereby reducing malondialdehyde (MDA) and hydrogen peroxide (H_2_O_2_) within plant tissues [26]. Similar performance has been observed in garlic [27] and wheat sprouts [28] under NaCl stress with Se enrichment pretreatment.

In recent years, emerging studies have focused on the biological effects and application of selenium nanoparticles (SeNPs). Nagdalian et al. [29] reported that SeNPs may possess higher bioavailability and lower phytotoxicity. Moreover, SeNPs have been demonstrated to enhance the accumulation of polyphenols and GABA in foxtail millet sprouts, compared to Na_2_SeO_3_ [18]. While SeNPs still face practical bottlenecks such as complex synthesis procedure, poor colloidal stability, and aggregation tendency [30]. In contrast, Na_2_SeO_3_ is a cost-effective Se source for Se enrichment with a simple and practical process during seed germination.

### 3.2. Effects of Na_2_SeO_3_ on Phenolic and GABA Content of Foxtail Millet Sprouts Under NaCl Stress

As shown in Figure 1C, with the increase in salt concentration, the TPC of foxtail millet sprouts increased first and then decreased. Meanwhile, after being pretreated with Na_2_SeO_3_, the TPC of foxtail millet sprouts further increased under NaCl concentration of 100 mM. Appropriate salt stress was conducive to metabolism and synthesis of diverse phenolic compounds [31]. These results were consistent with the findings of Lan et al. [28], who reported that Na_2_SeO_3_ enhances polyphenol levels of wheat seedlings under salt stress.

As shown in Figure 1D, NaCl stress induced a dose-dependent response in GABA accumulation: initially increasing under low salinity, reaching a maximum at approximately 100 mM, and subsequently declining at higher concentrations, reflecting the dual effect of salt stress on GABA metabolism. Notably, Na_2_SeO_3_ pretreatment significantly boosted GABA content under salt stress. Specifically, the GABA level in sprouts presoaked with Na_2_SeO_3_ solution was 16.4% higher than that in sprouts treated with 100 mM NaCl alone. It is believed that abiotic stress would lead to the synthesis and accumulation of GABA in plant tissue [32]. Wu et al. [33] reported that combining ultrasound with salt stress significantly increased GABA in germinated brown glutinous rice, and they hypothesized that this increase might be related to the enhanced GAD enzyme activity and the accelerated protein hydrolysis, leading to increased free amino acids, including glutamic acid, a precursor of GABA.

### 3.3. Effects of Germination Conditions on Phenolic and GABA Content of Foxtail Millet Sprouts

#### 3.3.1. Na_2_SeO_3_ Concentration

**Figure 2:**
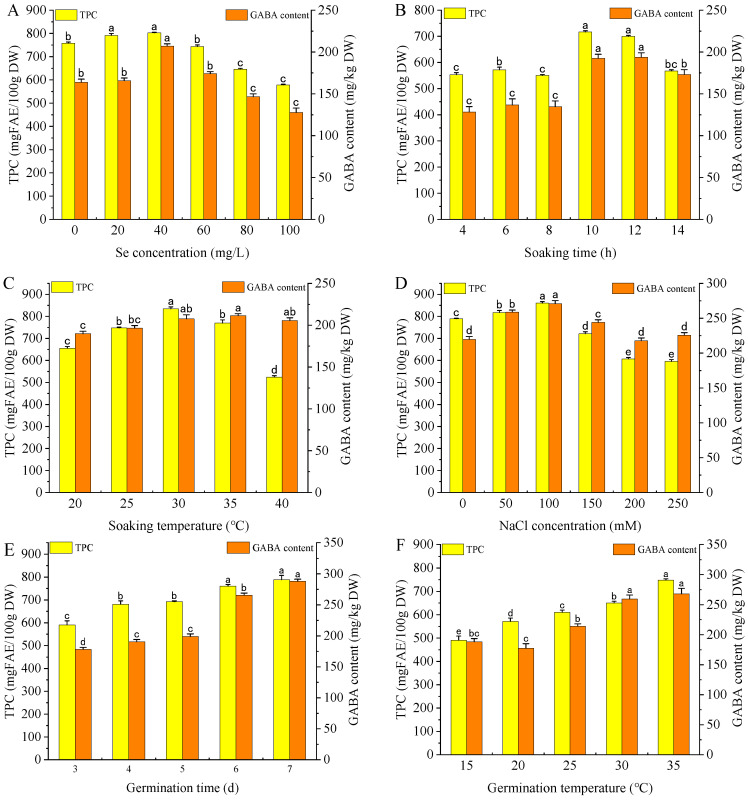
Effects of the germination conditions on TPC and GABA content of foxtail millet sprouts. (**A**) Na_2_SeO_3_ concentration, (**B**) soaking time, (**C**) soaking temperature, (**D**) NaCl concentration, (**E**) germination time, (**F**) germination temperature. Values are presented as mean ± SD. Values with different lowercase letters in the same index differ significantly (*p* < 0.05). Note, Se concentration: Na_2_SeO_3_ presoaking concentration.

The effects of Na_2_SeO_3_ presoaking concentration on the TPC and GABA content of foxtail millet sprouts are presented in Figure 2A. The TPC increased and then decreased with increasing Na_2_SeO_3_ presoaking concentration, peaking with 802.61 mg/100 g at 40 mg/L. The GABA content exhibited a parallel variation pattern to the TPC, with GABA reaching the maximal value of 207.13 mg/kg DW under 40 mg/L Na_2_SeO_3_. Low-concentration Na_2_SeO_3_ pretreatment promotes plant growth by modifying specific physiological and biochemical processes, thereby enhancing NaCl stress tolerance during germination [34]. However, high concentrations of Na_2_SeO_3_ solution can induce phytotoxicity in plants [35]. The highest TPC and GABA content were observed at the optimal Na_2_SeO_3_ concentration of 40 mg/L. Consequently, Na_2_SeO_3_ concentrations of 20, 40, and 60 mg/L were selected as the levels for the following response surface experiment.

#### 3.3.2. Na_2_SeO_3_ Presoaking Time

As seen in Figure 2B, TPC and GABA levels reached a maximum at 10 h soaking, followed by a gradual decline. As the soaking time prolongs, the cellular structure is disrupted, leading to dissolution of water-soluble substances and loss of glutamic acid required for GABA production [36]. Meanwhile, prolonged soaking may cause leaching of soluble polar phenolic components and potential degradation of polyphenols, resulting in a decrease in TPC. Based on the experimental results, 8, 10, and 12 h were selected as the levels for the response surface experiment.

#### 3.3.3. Na_2_SeO_3_ Presoaking Temperature

As shown in Figure 2C, the TPC and GABA content increased with soaking temperature from 20 to 30 °C, while TPC decreased slightly beyond 30 °C. It is presumed that an appropriate increase in soaking temperature may accelerate metabolic processes in seeds, for example, protein hydrolysis, and provide more amino acids as precursors (e.g., glutamic acid and phenylalanine) for the biosynthesis of GABA and polyphenols [37]. Studies have shown that excessively high soaking temperatures will destroy the internal structure of the seeds, leading to the leaching of water-soluble phenolic compounds [38]. Therefore, soaking temperatures of 25, 30 and 35 °C were selected for the subsequent experiments.

#### 3.3.4. NaCl Concentration

Figure 2D depicts that TPC and GABA accumulation in sprouts significantly increased with NaCl concentration from 0 to 100 mM (*p* < 0.05). When the NaCl concentration exceeded 100 mM, the synthesis of polyphenols was inhibited. Studies have reported that lower concentrations of NaCl stress promoted phenolic and GABA synthesis during legume seed germination [32,39]. Conversely, it has been reported that higher NaCl stress inhibited sprout growth and reduced polyphenol synthesis [40]. Consequently, the NaCl concentrations of 50, 100, and 150 mM were selected for the response surface experiment.

#### 3.3.5. Germination Time

As shown in Figure 2E, with the extension of germination time, the accumulation of phenolics and GABA in foxtail millet sprouts was significantly increased, which was consistent with the phenomenon observed in germinated highland barley [41] and brown rice [42]. However, germination beyond 3 days may induce morphological transition from sprouts to foliar structures, thereby compromising the edibility of cereal sprouts, as shown in Figure S1. Therefore, 3 days was assigned as the optimal germination time to balance bioactive accumulation and good palatability of millet sprouts.

#### 3.3.6. Germination Temperature

Figure 2F shows the variation in TPC and GABA contents with germination temperature. Within the germination temperature range of 15–35 °C, the levels of TPC and GABA in foxtail millet sprouts also increased correspondingly. However, high germination temperature accelerated sprout development, rendering sprouts unsuitable for consumption when exceeding 30 °C, as shown in Figure S1. Therefore, the germination temperature was finally selected as 30 °C.

### 3.4. Fitting the Model

**Figure 3:**
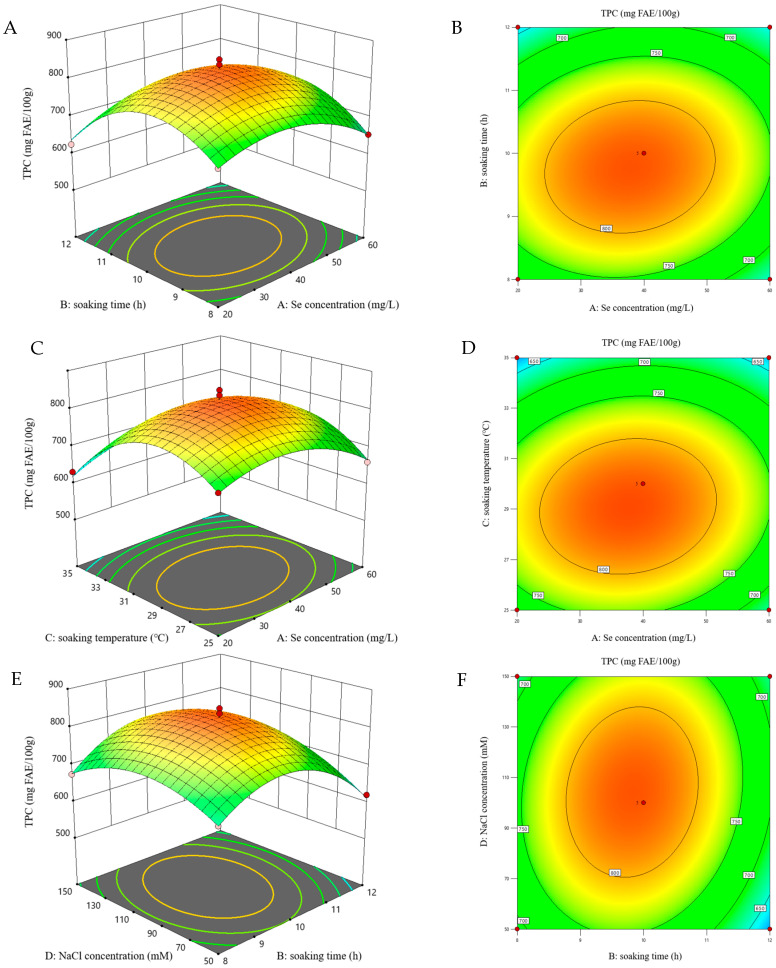
3D response surface and contour plots for TPC of foxtail millet sprouts: Interactive effects of Na_2_SeO_3_ concentration with soaking time (**A**,**B**), Na_2_SeO_3_ concentration with soaking temperature (**C**,**D**), and soaking time with NaCl concentration (**E**,**F**). Note, Se concentration: Na_2_SeO_3_ presoaking concentration.

**Figure 4:**
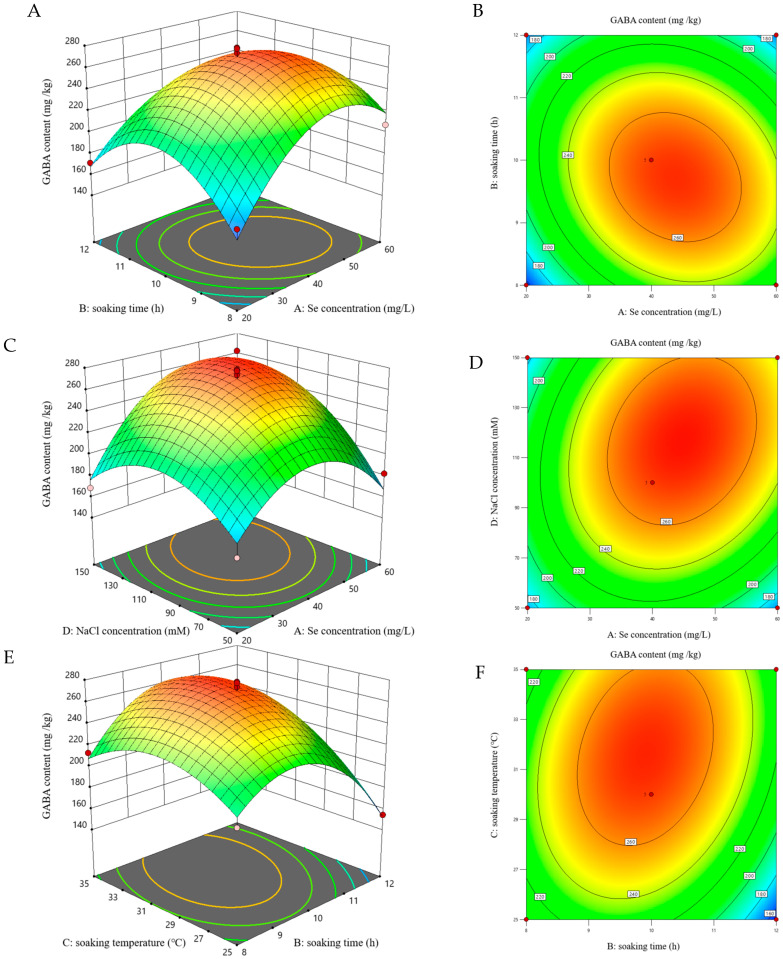
3D response surface and contour plots for GABA of foxtail millet sprouts: Interactive effects of Na_2_SeO_3_ concentration with soaking time (**A**,**B**), Na_2_SeO_3_ concentration with NaCl concentration (**C**,**D**), and soaking time with soaking temperature (**E**,**F**). Note, Se concentration: Na_2_SeO_3_ presoaking concentration.

Response surface methodology (RSM) accurately simulates and predicts system responses through polynomial functions and experimental designs [43], thereby maximizing polyphenol and GABA levels through optimized germination conditions for foxtail millet sprouts. TPC (Y_1_) and GABA (Y_2_) levels under various germination conditions were evaluated in 29 experimental runs, with results shown in Table 2. To evaluate the quadratic polynomial model for yield, t-tests were performed to assess the significance of individual terms, and ANOVA was used to test the overall model fit. As shown in Table 3, variable significance is reflected by the *p*-value, with smaller values indicating a stronger influence on the results, while the relative significance of each factor for TPC and GABA yield was evaluated using F-values. Figure 3 and Figure 4 present the 3D plots and their corresponding contour plots for the interaction terms that have significant effects on the TPC and GABA content, respectively. Surface steepness in the 3D plot indicates the strength of the two-factor interaction. The steeper the surface, the more significant the effect of their combined influence on the response value. On the contour plot, the values on each curve are the same. The ellipse represents a significant interaction effect between these two factors, and the larger the ellipse, the greater the influence of the interacting factors. Additionally, the transition of colors from blue through green to red indicates progressively higher values.

#### 3.4.1. TPC

The regression model examining Na_2_SeO_3_ concentration effects on TPC in stressed foxtail millet sprouts demonstrated extremely high significance (*p* < 0.0001), as shown in Table 3. This exceptional significance was evidenced by a high coefficient of determination (R^2^) of 0.9817 and an adjusted coefficient of determination (R^2^adj) of 0.9633 with a negligible difference (<0.2), along with an insignificant lack of fit (*p* > 0.05). The statistical analysis indicated that the model can be employed to analyze and predict the relationship between TPC and related variables. The four factors had significant effects (*p* < 0.05) on TPC, specifically, the Na_2_SeO_3_ presoaking concentration (A), soaking time (B), soaking temperature (C), and NaCl stress concentration (D). Additionally, the corresponding secondary terms (A^2^, B^2^, C^2^, D^2^) also had significant effects on TPC (*p* < 0.05). Furthermore, the significant effects (*p* < 0.05) on TPC were observed for the AB, AC and BD interaction terms. Subsequently, non-significant variables (*p* > 0.05) were excluded, and predicted values of TPC were calculated via Equation (2).

(2) $$\mathrm{TPC}=826.26-11.94A-20.00B-35.75C+12.10D+16.87\mathrm{AB}+15.57\mathrm{AC}+17.26\mathrm{BD}-61.76A^{2}-102.37B^{2}-105.65C^{2}-61.60D^{2}$$

The response surface and contour plots of interaction terms with significant influence are shown in Figure 3. These plots clearly illustrate the interaction between the two independent variables. As the values of the independent variables increased, the TPC gradually increased and reached a maximum, then decreased. For example, when the Na_2_SeO_3_ concentration was fixed, the TPC increased gradually as the soaking time was extended from 8.0 to 9.8 h. However, further extending the soaking time led to a decrease in TPC.

#### 3.4.2. GABA Content

Table 3 shows that the regression model for GABA response exhibited high significance, with a *p*-value of less than 0.001. With an F-value of 2.64 and a *p*-value exceeding 0.05 for the lack of fit, the regression model was confirmed to be statistically significant [38]. The R^2^ and R^2^adj values were 0.9363 and 0.8727, respectively, with a difference of less than 0.2, suggesting a good fit and reliable prediction of GABA content from the relevant variables. The correlation between GABA content and the four variables was described by a second-order polynomial model, as shown in Equation (3).

(3) $$\mathrm{GABA}=270.18+15.22A-9.91B+17.84C+19.73D-15.74\mathrm{AB}+17.84\mathrm{AD}+16.37\mathrm{BC}-45.32A^{2}-47.51B^{2}-26.19C^{2}-35.13D^{2}$$

As shown in Figure 4, the 3D response surface plots indicate favorable interactions among several pairs of factors. Specifically, good interactions between Na_2_SeO_3_ presoaking concentration and soaking time, Na_2_SeO_3_ presoaking concentration and NaCl concentration, and soaking temperature and soaking time were observed. Moreover, all these interaction factors were significant (*p* < 0.05). Under a suitable low concentration range, the GABA level increased in response to higher concentrations of presoaking Na_2_SeO_3_ and NaCl stress. Nevertheless, with a further increase in NaCl concentration, maintaining a high GABA level demanded a corresponding elevation of Na_2_SeO_3_ concentration. Notably, an excessive increase in NaCl concentration ultimately inhibited GABA production.

#### 3.4.3. RSM Model Validation

The optimal germination conditions obtained from the model of RSM results were as follows: 41.823 mg/L for Na_2_SeO_3_ concentration, 9.818 h for soaking time, 30.037 °C for soaking temperature, and 110.057 mM for NaCl concentration. When these conditions were combined, the model predicted the maximum TPC and GABA values to be 824.90 mg/100g DW and 274.77 mg/kg DW, respectively. Considering operational feasibility and convenience, the actual process parameters were set to: 42 mg/L Na_2_SeO_3_, 9.8 h soaking time, 30 °C soaking temperature, and 110 mM NaCl. The results of three parallel experiments conducted using the adjusted process parameters are shown in Table 4. Under the optimal conditions, the total phenolics and GABA contents in Se-enriched foxtail millet sprouts reached 837.22 mg/100g DW and 281.68 mg/kg DW, respectively, which closely matched the predicted values, thereby validating the adequacy of the model Equation (2) and model Equation (3).

### 3.5. Phenolic, GABA, and Se Contents Before and After Optimization

Table 4 compares the contents of GABA and phenolic compounds levels of sprouts under NaCl stress (pre-optimized) and Na_2_SeO_3_ presoaking combined with NaCl stress (post-optimized). The optimized germination conditions significantly increased TPC by 17.1% and GABA by 47.7% in foxtail millet sprouts compared to pre-optimization levels (*p* < 0.05). Presoaking treatment with Na_2_SeO_3_ under NaCl stress promoted the accumulation of polyphenols and GABA. In addition, consistent with our previous identification results [22], the phenolic composition of foxtail millet sprouts showed no significant changes before and after optimization, as shown in Figures S2 and S3. This indicates that the Na_2_SeO_3_ presoaking treatment and NaCl stress treatment have no significant impact on the phenolic profile of foxtail millet sprouts. For the individual polyphenolic compounds in free form, except for 4-*p*-coumaroylquinic acid, the levels of the remaining phenolic constituents increased significantly (*p* < 0.05). It is worth mentioning that two hydroxycinnamic acid derivatives, *N*-*p*-coumaroylserotonin and *N*-feruloylserotonin, increased by 3.34 and 1.76 times, respectively. The synergistic effect between the serotonin backbone and phenolic acyl groups of *N*-*p*-coumaroylserotonin and *N*-feruloylserotonin provides them with stronger antioxidant, anti-inflammatory and neuroprotective activities [44]. Meanwhile, the contents of *p*-hydroxybenzoic acid, 3-*p*-coumaroylquinic acid, *trans*-ferulic acid and 3,7-dimethylquercetin increased by 2.64, 2.94, 0.82 and 2.53 times, respectively. The contents of the major bound polyphenolic compounds, *trans*-ferulic acid and *trans*-*p*-coumaric acid, increased by 2.28 and 6.39 times, respectively. Overall, the individual phenolic level of foxtail millet sprouts was significantly upregulated by Se enrichment under salt stress.

The United States, the European Union, and China define the criteria for “selenium-enriched foods” as Se content not lower than 0.11 mg/kg, 0.165 mg/kg, and 0.18 mg/kg, respectively [45]. Under optimized germination conditions, the Se-enriched foxtail millet sprouts achieved a total Se content of 14.74 mg/kg dry weight and organic Se content of 12.02 mg/kg dry weight, which are far higher than the standard values for Se-enriched foods. The high Se level of the foxtail millet sprouts makes them particularly suitable to be incorporated into various food products to achieve desired Se fortification. From the perspective of dietary safety, the tolerable upper intake level of Se for adults is approximately 400 μg per day [46]. Based on the total Se content of optimized foxtail millet sprouts in this study, the daily intake of such Se-enriched sprouts should be limited to 27.14 g dry weight for adults.

## 4. Conclusions

The germination conditions for foxtail millet sprouts were optimized by using RSM based on the TPC and GABA content, and the optimal germination parameters were 42 mg/L Na_2_SeO_3_, 9.8 h soaking time, 30 °C soaking temperature, and 110 mmol/L NaCl solution. Under these parameters, the TPC of foxtail millet sprouts was 837.22 mg FAE/100 g, and the GABA content reached 281.68 mg/kg, which were in good agreement with the predicted values of 824.90 mg/100 g and 274.77 mg/kg, respectively. The majority of free and bound individual phenolic compounds in the foxtail millet sprouts were also significantly increased (*p* < 0.05), especially the bound *trans*-*p*-coumaric acid, which increased by 6.39-fold. In addition, the total Se content (14.74 mg/kg DW) and organic Se content (12.02 mg/kg DW) significantly exceeded the standard for Se-enriched foods. These results indicate that foxtail millets presoaked with Na_2_SeO_3,_ combined with germination under NaCl stress, could simultaneously increase both Se and functional components in foxtail millet sprouts, providing a practical technology for developing high-value-added Se-enriched cereal sprouts for the food industry. The main limitation of this study is that the key enzyme activities involved in the polyphenol and GABA metabolic pathways were not investigated. The key enzyme activities and gene expression levels could be included in our future research, together with biomass and germination index, to fully elucidate the potential mechanisms of polyphenol and GABA accumulation by Se-enriched germination under salt stress.

## Figures and Tables

**Table 1:** Variables and their levels in the experimental design.

Independent Variables	Code Units	Coded Variable Level		
		−1	0	1
**Na_2_SeO_3_ concentration (mg/L)**	A	20	40	60
**Soaking time (h)**	B	8	10	12
**Soaking temperature (°C)**	C	25	30	35
**NaCl concentration (mM)**	D	50	100	150

**Table 2:** RSM experimental design and results.

Design Point	A (mg/L)	B (h)	C (°C)	D (mM)	GABA Content (mg/kg)	TPC (mg/100g)
1	60	10	25	100	187.46 ± 3.22	660.66 ± 2.81
2	60	12	30	100	150.40 ± 2.59	635.97 ± 1.73
3	40	10	35	150	230.84 ± 4.76	630.57 ± 3.63
4	20	12	30	100	171.40 ± 5.45	625.01 ± 3.18
5	40	8	35	100	213.28 ± 8.05	611.79 ± 4.24
6	40	10	30	100	278.18 ± 9.31	810.92 ± 5.85
7	40	8	30	50	184.02 ± 3.90	687.27 ± 3.70
8	40	12	30	150	198.05 ± 8.12	677.53 ± 8.10
9	40	10	35	50	191.13 ± 7.98	579.62 ± 3.21
10	60	10	30	50	182.48 ± 3.72	683.44 ± 2.85
11	40	12	30	50	152.24 ± 3.28	621.01 ± 2.74
12	60	10	35	100	232.72 ± 8.25	631.62 ± 2.80
13	40	10	30	100	267.56 ± 6.53	807.97 ± 6.33
14	20	10	35	100	210.12 ± 8.08	631.62 ± 2.72
15	40	10	25	50	183.86 ± 4.70	701.18 ± 3.13
16	40	10	30	100	273.55 ± 3.71	850.40 ± 2.82
17	40	10	30	100	254.37 ± 4.57	825.01 ± 4.56
18	40	10	25	150	215.14 ± 6.92	701.36 ± 6.74
19	20	10	25	100	164.32 ± 4.72	722.92 ± 4.52
20	20	8	30	100	165.59 ± 2.98	710.40 ± 1.74
21	20	10	30	50	160.18 ± 4.01	703.27 ± 1.52
22	20	10	30	150	168.56 ± 5.57	726.23 ± 2.21
23	40	12	35	100	236.99 ± 5.69	572.49 ± 4.82
24	60	10	30	150	262.22 ± 9.31	710.57 ± 4.20
25	60	8	30	100	207.56 ± 3.55	653.88 ± 3.55
26	40	8	30	150	215.80 ± 8.24	674.75 ± 6.86
27	40	8	25	100	195.99 ± 2.02	667.27 ± 1.91
28	40	12	25	100	154.23 ± 6.39	633.36 ± 3.99
29	40	10	30	100	277.26 ± 2.86	837.01 ± 1.05

**Table 3:** ANOVA of the regression model.

Source	TPC		GABA Content	
	F-Value	*p*-Value	F-Value	*p*-Value
Model	53.52	<0.0001 **	14.71	<0.0001 **
A	8.24	0.0123 *	13.39	0.0026 **
B	23.12	0.0003 **	5.67	0.0319 *
C	73.89	<0.0001 **	18.39	0.0007 **
D	8.47	0.0114 *	22.49	0.0003 **
AB	5.48	0.0345 *	4.78	0.0464 *
AC	4.67	0.0485 *	0.00	0.9855
AD	0.02	0.8869	6.13	0.0267 *
BC	0.04	0.8543	5.16	0.0394 *
BD	5.74	0.0311 *	0.24	0.6339
CD	3.11	0.0998	0.09	0.7743
A^2^	119.17	<0.0001 **	64.18	<0.0001 **
B^2^	327.42	<0.0001 **	70.50	<0.0001 **
C^2^	348.76	<0.0001 **	21.43	0.0004 **
D^2^	118.58	<0.0001 **	38.56	<0.0001 **
Lack of Fit	0.52	0.8197	2.64	0.1809
R^2^	0.9817		0.9363	
Adj R^2^	0.9633		0.8727	
Pred R^2^	0.9280		0.6684	

* represents *p* < 0.05, ** represents *p* < 0.01.

**Table 4:** Phenolics, GABA, and Se in foxtail millet sprouts: comparison before and after optimization.

	Phenolic Compounds	Individual Phenolic Content (mg/kg DW)	
		Before Optimization	After Optimization
Free	*p*-hydroxybenzoic acid	2.50 ± 0.03 ^b^	9.09 ± 0.14 ^a^
	3-*p*-coumaroylquinic acid	2.34 ± 0.04 ^b^	9.23 ± 0.08 ^a^
	*p*-hydroxybenzaldehyde	0.66 ± 0.01 ^b^	1.11 ± 0.02 ^a^
	*N*-(*p*-coumaroyl) serotonin	3.84 ± 0.03 ^b^	16.66 ± 0.98 ^a^
	*N*-feruloylserotonin	3.44 ± 0.03 ^b^	9.48 ± 0.48 ^a^
	4-*p*-coumaroylquinic acid	14.37 ± 0.19 ^a^	14.05 ± 0.08 ^a^
	Feruloylquinic acid	24.39 ± 0.33 ^b^	25.59 ± 0.51 ^a^
	*trans*-ferulic acid	6.56 ± 0.15 ^b^	11.96 ± 0.42 ^a^
	3,7-dimethylquercetin	8.14 ± 0.08 ^b^	28.75 ± 0.95 ^a^
Bound	*p*-hydroxybenzaldehyde	2.24 ± 0.08 ^b^	13.89 ± 0.25 ^a^
	Vanillic acid	1.59 ± 0.03 ^b^	9.27 ± 0.41 ^a^
	Syringic acid	1.66 ± 0.04 ^b^	9.47 ± 0.19 ^a^
	*trans*-*p*-coumaric acid	44.96 ± 0.23 ^b^	332.38 ± 10.48 ^a^
	*cis*-*p*-coumaric acid	1.57 ± 0.01 ^b^	7.23 ± 0.17 ^a^
	*trans*-ferulic acid	483.51 ± 3.83 ^b^	1584.73 ± 22.75 ^a^
	*cis*-ferulic acid	19.25 ± 0.4 ^b^	63.51 ± 1.55 ^a^
	TPC (mg FAE/100g DW)	714.99 ± 12.76 ^b^	837.22 ± 17.73 ^a^
	GABA content (mg/kg DW)	190.71 ± 10.24 ^b^	281.68 ± 8.00 ^a^
	Total selenium content (mg/kg DW)	—	14.74 ± 0.37
	Organic selenium content (mg/kg DW)	—	12.02 ± 0.43

Note: —: not determined. Different letters in same row represent significant differences (*p* < 0.05).

## Data Availability

The original contributions presented in this study are included in the article/Supplementary Material. Further inquiries can be directed to the corresponding author.

## Supplementary Materials

The following supporting information can be downloaded at: https://www.mdpi.com/article/10.3390/foods15101778/s1, Figure S1: The growth morphology of foxtail millet sprouts under different germination conditions; Figure S2. UPLC chromatograms of free phenolic extract from foxtail millet sprouts before (A) and after (B) optimization; Figure S3. UPLC chromatograms of bound phenolic extract from foxtail millet sprouts before (A) and after (B) optimization.

## Abbreviations

The following abbreviations are used in this manuscript:

RSM	Response surface methodology
BBD	Box–Behnken design
TPC	Total phenolic content
GABA	γ-aminobutyric acid
Se	Selenium
UPLC-MS/MS	Ultrahigh-performance liquid chromatography tandem mass spectrometry
FAE	Ferulic acid equivalents
DW	Dry weight of sample
