# Supplementary material for: Presoaking with Sodium Selenite Promotes Accumulation of Polyphenols and GABA in Foxtail Millet Sprouts Under NaCl Stress
Source: Foods. 2026 May 18;15(10):1778. doi: 10.3390/foods15101778 (PMC13205534; doi:10.3390/foods15101778)
Supplement: Supplementary file 1 [file foods-15-01778-s001.zip › foods-4305131-supplementary.pdf]

Presoaking with Sodium Selenite Promotes Accumulation of Polyphenols and GABA in Foxtail Millet Sprouts under NaCl Stress

Huiying Fu [1], Shuaiduo Sun [1, 2], Yaoxi Liu [2], Guowei Man [1], Junjie Hao [3], Jinle Xiang [1, 4*]

[1] Henan University of Science & Technology, Faculty of Food & Bioengineering, Luoyang, Henan, 471023, China

[2] Henan Forestry Vocational College, Department of Tourism and Food, Luoyang, Henan, 471000, China

[3] Institute of Plant Protection, Henan Academy of Agricultural Sciences, Zhengzhou, 450002, China

[4] Henan University of Science & Technology, Henan Key Laboratory of Agricultural Product Processing Technology, Luoyang, Henan, 471023, China

* Corresponding author. Address: Faculty of Food & Bioengineering, Henan University of Science & Technology, Luoyang, Henan, 471023, China.

Tel.: +86-15237916981. ORCID IDs: orcid.org/0000-0001-5652-117X. E-mail address: xjl5013@haust.edu.cn

Huiying Fu & Shuaiduo Sun contributed equally to this research.

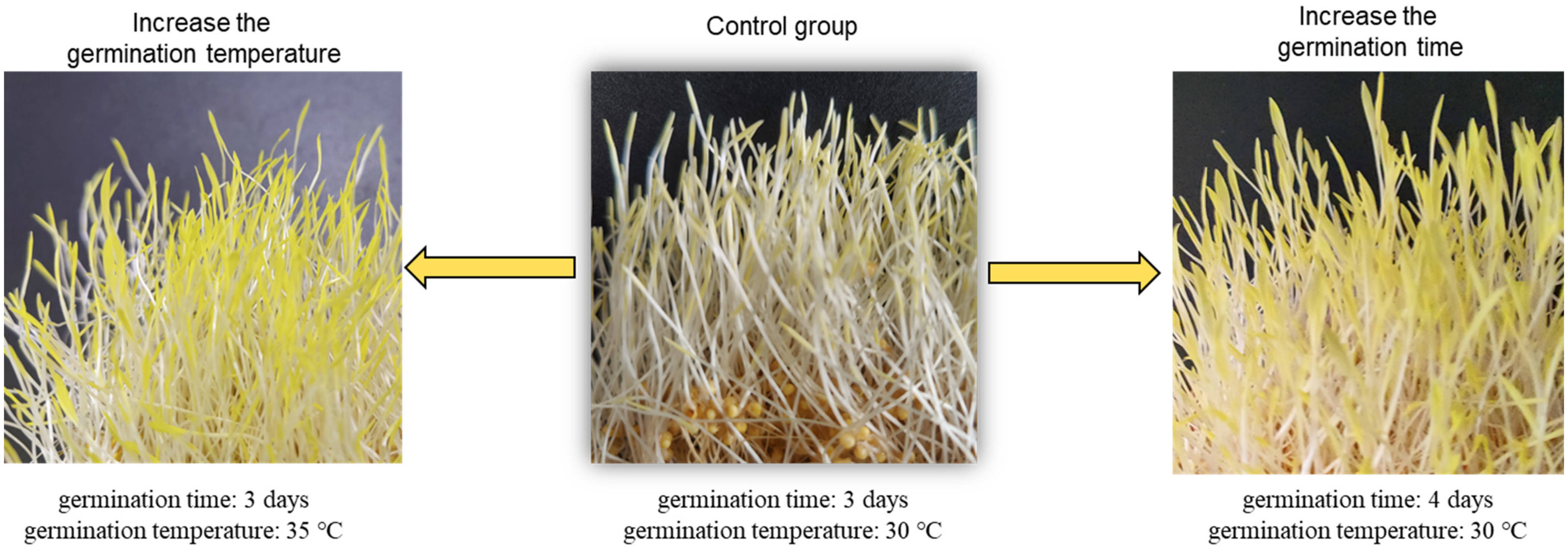

Figure S1. The growth morphology of foxtail millet sprouts under different germination conditions.

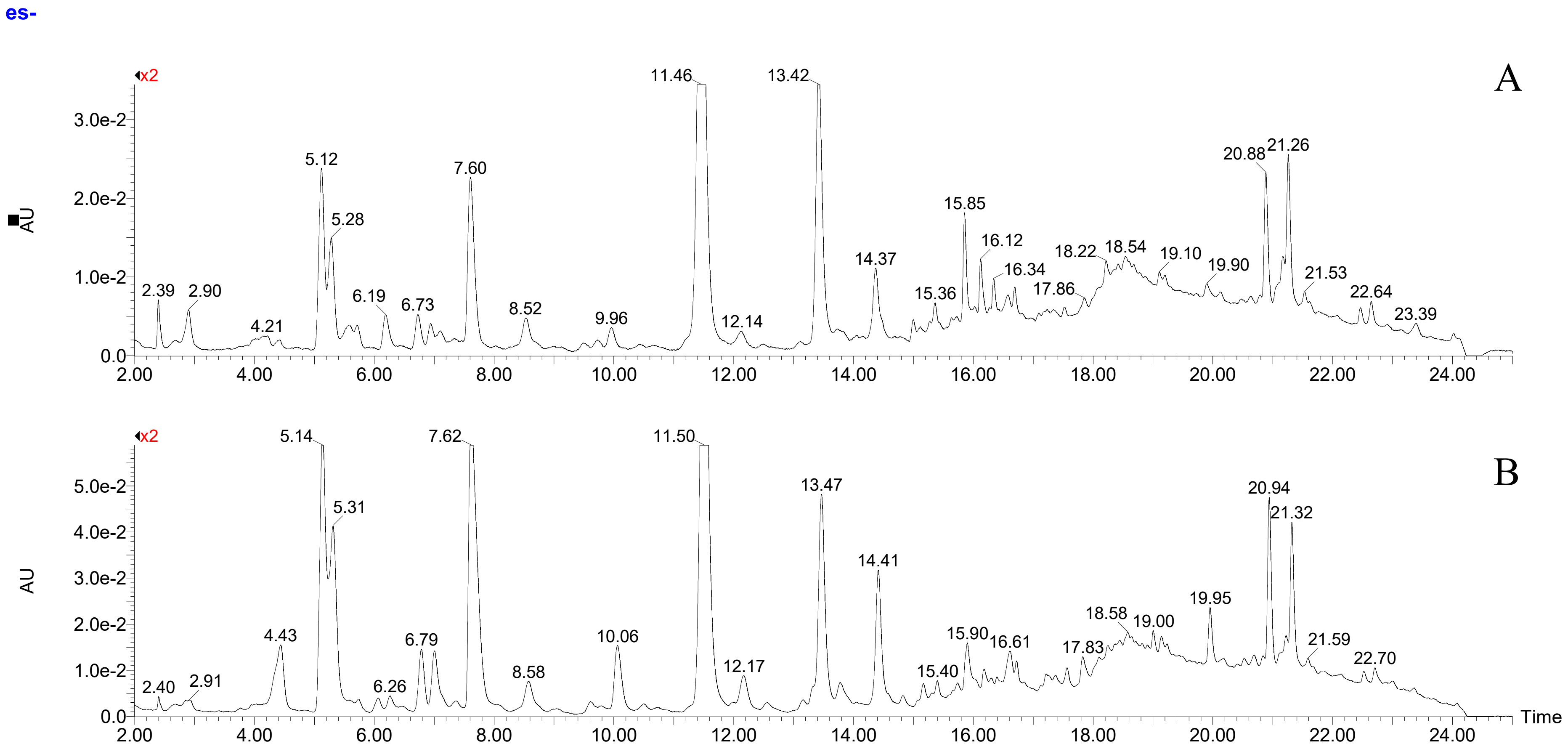

Figure S2. UPLC chromatograms of free phenolic extract from foxtail millet sprouts before (A) and after (B) optimization. Note: 5.12: *p*-hydroxybenzoic acid; 5.28: 3-*p*-coumaroylquinic acid; 6.73: *p*-hydroxybenzaldehyde; 7.60: *N*-(*p*-coumaroyl) serotonin; 9.96: *N*-feruloylserotonin; 11.46: 4-*p*-coumaroylquinic acid; 13.42: Feruloylquinic acid; 14.37: *trans*-ferulic acid; 21.26: 3,7-dimethylquercetin.

es-

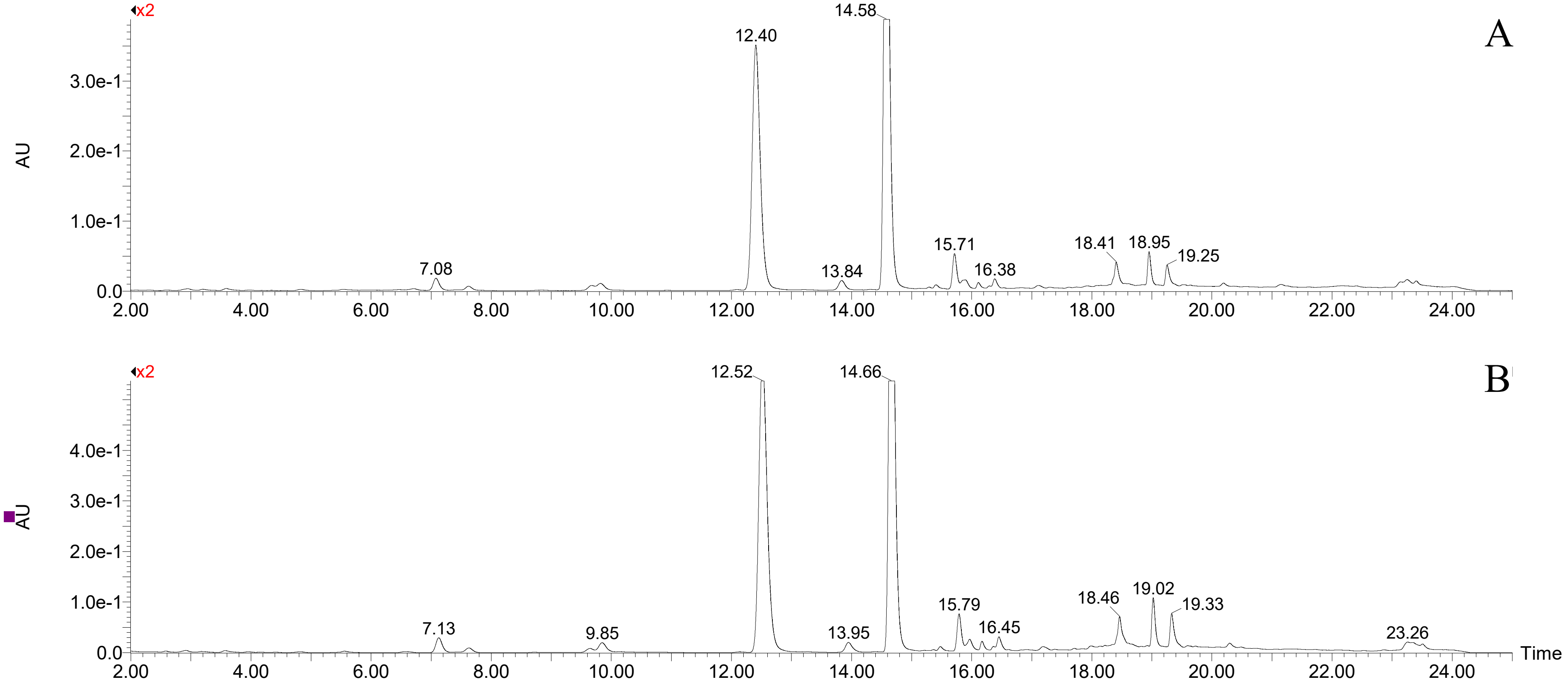

Figure S3. UPLC chromatograms of bound phenolic extract from foxtail millet sprouts before (A) and after (B) optimization. Note: 7.08: *p*-hydroxybenzaldehyde; 7.82: Vanillic acid; 9.80: Syringic acid; 12.40: *trans*-*p*-coumaric acid; 13.84: *cis*-*p*-coumaric acid; 14.58: *trans*-ferulic acid; 15.71: *cis*-ferulic acid.
